# Heterotrophic bacterial production and extracellular enzymatic activity in sinking particulate matter in the western North Pacific Ocean

**DOI:** 10.3389/fmicb.2012.00379

**Published:** 2012-10-23

**Authors:** Namiha Yamada, Hideki Fukuda, Hiroshi Ogawa, Hiroaki Saito, Masahiro Suzumura

**Affiliations:** ^1^Research Institute for Environmental Management Technology, National Institute of Advanced Industrial Science and TechnologyTsukuba, Japan; ^2^International Coastal Research Center, Atmosphere and Ocean Research Institute, The University of TokyoOtsuchi, Iwate, Japan; ^3^Atmosphere and Ocean Research Institute, The University of TokyoKashiwa, Chiba, Japan; ^4^Tohoku National Fisheries Research Institute, Fisheries Research AgencyShiogama, Miyagi, Japan

**Keywords:** sinking particulate matter, sediment trap, heterotrophic bacterial activity, extracellular enzyme activity, western North Pacific

## Abstract

Heterotrophic activities on sinking particulate matter (SPM) play an important role in SPM fluxes in the ocean. To demonstrate regional differences in heterotrophic activities on SPM, we measured heterotrophic bacterial production (HBP) in seawater (HBP_SW_) and SPM (HBP_SPM_) as well as potential extracellular enzyme activity (EEA) in SPM on a transect along 155°E in the western North Pacific Ocean in the subarctic (44°N), the Kuroshio Extension area (35°N), and the subtropical gyre (20°N). Depth-integrated HBP_SW_ from the surface to 500 m was comparable between the locations, whereas HBP_SPM_ at 44°N was substantially lower than at the other sites. We found the highest particulate organic carbon (POC) export flux and export efficiency to bathypelagic depths, and the lowest water temperatures, at 44°N. We found significant correlations between leucine aminopeptidase (LAPase) activity, β-glucosidase (BGase) activity, POC flux and particulate organic nitrogen flux. LAPase activity was two orders of magnitude higher than BGase activity, with a BGase:LAPase activity ratio of 0.027. There were no significant correlations between HBP and EEA in SPM except for lipase, and lipase activity was significantly correlated with temperature. We propose that hydrographic conditions are an important factor controlling heterotrophic bacterial activity and export efficiency of organic carbon to the deep ocean, as are the sources and abundance of SPM produced in the euphotic zone via primary production.

## INTRODUCTION

Physical and biogeochemical processes in the ocean are major regulators of atmospheric carbon dioxide (CO_2_), with the ocean particularly important as a sink for fossil fuel CO_2_. Therefore, understanding the biogeochemistry of carbon in the oceans is key to predicting and assessing the future evolution of climate. It is essential to quantify the processes that control the transport of carbon and nutrients that support marine primary production (PP) from the surface to the deep ocean. Sinking particulate matter (SPM) plays an important role in transporting and redistributing carbon and nutrients in the ocean as part of the “biological pump” (Volk and Hoffert, 1985; Boyd and Trull, 2007). It is estimated that 2–20% of PP is exported from the surface euphotic zone to mesopelagic depths (Boyd and Trull, 2007). In addition to gravitational sinking of particulate organic matter, the downward flux of dissolved organic matter (DOM) could play a more important role in carbon transport in the ocean’s interior than previously thought (Toggweiler, 1988; Hansell et al., 2009). In both SPM and DOM cycling, heterotrophic bacteria have been recognized as the major consumers and transformers of PP in ocean ecosystems (Karl et al., 1988; Fuhrman, 1992; Steinberg et al., 2008). Heterotrophic bacterial production (HBP) comprises 30% of PP integrated over the entire water column (Cole et al., 1988). Nagata et al. (2000) summarized that HBP at mesopelagic and bathypelagic depths accounts for 38–118% of sinking particulate organic carbon (POC).

Marine sinking particles provide potential “hot spots” for microbial decomposition of organic matter (Azam, 1998; Azam and Long, 2001). Because bacteria can only incorporate small molecules (<600 Da) via their cell-membrane permeases, macromolecules and particles must be broken down to monomers prior to their incorporation (Weiss et al., 1991). HBP in the ocean interior is apparently fueled by the enzymatic hydrolysis of organic matter in SPM to dissolved organic carbon (DOC), which is then remineralized to CO_2_ by the suspended, “free-living” bacteria in the ambient seawater as well as the pool of bacteria attached to the particles (Cho and Azam, 1988). Marine bacteria hydrolyze polymers and organic particles using extracellular enzymes, both cell surface-bound and those released into the ambient seawater (Azam and Malfatti, 2007; Nagata, 2008 and references cited there in). Extracellular enzyme activity (EEA) has previously been examined in various marine particles collected from mesopelagic environments, including suspended particulate matter (Hoppe et al., 1993), marine snow (Karner and Herndl, 1992; Smith et al., 1992), and sinking particles collected by sediment traps (Huston and Deming, 2002; Taylor et al., 2009). These studies measured the activities of hydrolytic enzymes such as proteases, lipases, chitinases, and glucosidases, which catalyze chemical bond cleavage in protein, lipid, and polysaccharide macromolecules, and phosphatases, which release phosphate.

In their comprehensive sediment trap study in the North Pacific Ocean, Buesseler et al. (2008) examined how physical, chemical, and biological conditions impact the transport efficiency of SPM at contrasting sites in the subarctic northwest Pacific Ocean and in the North Pacific subtropical gyre (NPSG) near Hawaii. They found differences between the two sites and identified heterotrophic degradation as one of the important processes in the transport. Steinberg et al. (2008) observed difference of depth-integrated bacterial carbon demand (BCD) and respiration in water column for loss of sinking POC flux between the same two sites. In this study, we aimed to further investigate differences in SPM transport efficiency across different geographic regions and identify variables which contributed to observed differences. We used a free-drifting sediment trap system to investigate SPM flux, HBP in seawater (HBP_SW_) and SPM (HBP_SPM_), and EEA in SPM at three locations with a large geographic variation, from the subarctic to subtropical western North Pacific Ocean. We measured EEA on SPM of four hydrolytic enzymes: leucine aminopeptidase (LAPase), β-glucosidase (BGase), lipase, and alkaline phosphatase (APase). We also examined the relative importance of the export flux of SPM and heterotrophic bacterial activity in the carbon biogeochemical cycle in the areas studied.

## MATERIALS AND METHODS

### STUDY SITES

**FIGURE 1:**
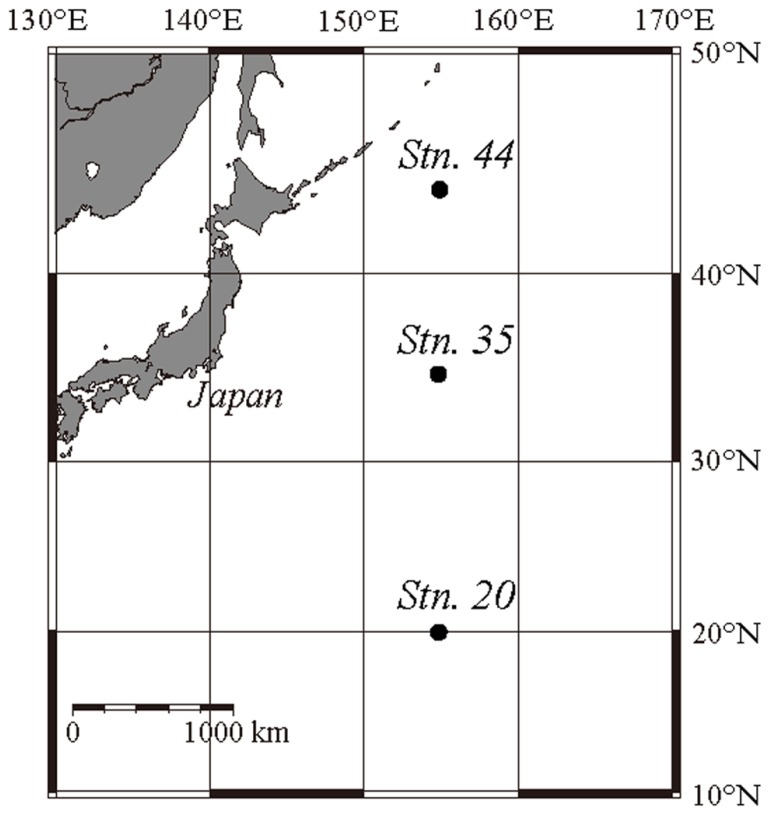
**Sites of sediment trap experiments in the western North Pacific Ocean**.

Samples were collected from the western North Pacific Ocean during cruise KH08-2 (Leg 2) on R/V *Hakuho-Maru* from August 23 to September 16, 2008. The sampling stations were located adjacent to the northwest Pacific subarctic gyre (station 44; 44°N), in the Kuroshio Extension area (station 35; 35°N), and in the NPSG (station 20; 20°N), all along longitude 155°E (**Figure 1**). The dates of sampling and sediment trap experiments at each station are shown in **Table 1**.

**Table 1:** Sediment trap deployments and target assays^a^ from trap tubes in this study.

Station	Date of deployment	Depth (m)	Trap tubes				
			#2	#3	#4	#5	#7
44	August 25, 2008	50	CN	SPM	SPM	—^b^	BAM
		200	CN	SPM	SPM	—	BAM
		500	CN	SPM	SPM	—	BAM
35	August 29, 2008	100	CN	CN	SPM	SPM	BAM
		200	CN	—	CN	SPM	BAM
		500	CN	CN	SPM	SPM	BAM
20	September 5, 2008	100	CN	CN	SPM	SPM	BAM
		200	CN	CN	—	SPM	BAM
		500	CN	CN	SPM	SPM	BAM

a *CN, POC and PON fluxes; SPM, sinking particulate matter weighed for total mass flux estimates; BAM, biological activity measurements including TdR and EEA (see text)*.

b *Tube contents used for other studies*.

### BIOGEOCHEMICAL PARAMETERS IN WATER COLUMN

Hydrographic data were provided by a shipboard conductivity–temperature–depth, (CTD) profiler equipped with a carousel multi-sampling system. We obtained water-column depth profiles of dissolved nutrients including nitrate, phosphate, and silicate, chlorophyll *a* (Chl *a*), bacterial cell abundance (BA), and HBP. Dissolved nutrients were measured in seawater collected at 15 depths from the surface to 500 m. We measured nutrient concentrations spectrophotometrically using an autoanalyzer (AACS II, Bran+Luebbe, Norderstedt, Germany), based on the methods described by Strickland and Parsons (1972). The detection limits of the autoanalyzer were 0.05 μmol L^−1^ for nitrate, 0.03 μmol L^−1^ for phosphate, and 0.2 μmol L^−1^ for silicate. Chl *a* concentration at 12 depths in the upper 200 m was measured fluorometri- cally using *N*, *N*-dimethylformamide extraction (Suzuki and Ishimaru, 1990).

We determined BA and HBP_SW_ samples collected at six depths from the surface to 500 m. BA was determined based on the SYBR GOLD counting method as described by Shibata et al. (2006). HBP_SW_ was determined on board using the microcentrifuge method with the ^3^H-labeled leucine (^3^H-Leu) incorporation technique to measure protein production (Kirchman, 2001). ^3^H-Leu (Perkin Elmer Life and Analytical Science, Boston, MA, USA; final concentration 20 nmol L^−1^, specific activity 5.92 TBq mmol^−1^) was added to 1.5 mL aliquots of duplicate seawater samples and duplicate killed controls [killed by adding ice-cold trichloroacetic acid (TCA), final concentration 5% (w/v)]. Samples and controls were incubated in the dark at *in situ* temperature (±2°C) for 1–24 h. Incubations were ended by adding TCA to a final concentration of 5% (w/v). After microcentrifugation, the supernatant was removed and 1 mL of liquid-scintillation cocktail (Ultima Gold, Perkin Elmer, Waltham, MA, USA) was added to the TCA precipitates. The radioactivity was determined with a liquid scintillation counter (Wallac 1414 WinSpectral, GMI Inc., Ramsey, MN, USA). To estimate HBP_SW_, we used a factor of 1.5 kg-C (mol Leu)^−1^ to convert the ^3^H-Leu incorporation to carbon equivalents, assuming no isotopic dilution (Kirchman, 2001).

### SEDIMENT TRAP EXPERIMENTS

We deployed standard cylindrical multi-traps following the configuration of Knauer et al. (1979), with eight acrylic trap tubes (7.0 cm internal diameter × 62 cm length) mounted at each depth. The bottom part of the cylindrical trap was separable as a collection cup with an approximate volume of 260 mL. The traps were set vertically on the array line at three targeted depths of 50, 200, and 500 m at station 44, and 100, 200, and 500 m at stations 35 and 20. The upper deployment depths were chosen to be just under or near the bottom of the euphotic zone (46, 61, and 103 m at stations 44, 35, and 20, respectively). The euphotic zone was defined as the depth at which photosynthetically active radiation was 1% of the value just below the surface (light intensity data provided by Dr K. Suzuki of Hokkaido University, Japan; personal communication). The material collected in each trap tube was used for various chemical and biochemical analyses (**Table 1**).

Before deployment, all trap tubes except tube #7 on each array were filled with seawater that had been collected from 4 m below the surface at each station using the ship’s pump, pre-filtered through a 0.2-μm capsule cartridge filter (MCS-020-D10SR, Advantec, Tokyo, Japan) to minimize biological contamination, and mixed with sodium chloride to a final concentration of 4% (w/v) to create a density gradient. Tube #7 at each depth was used for collecting samples for measuring the activity of heterotrophic bacteria and extracellular enzymes on SPM, and was filled with seawater filtered as described above that was collected just before deployment from the depth corresponding to the target layer of trap deployment with a 12-L Niskin bottle. The arrays were attached to a buoy and allowed to drift freely for 24 h at station 44, and 48 h at stations 35 and 20.

Upon recovery, the traps were stored upright in the dark and left to settle for 1 h. After the contents had settled, the upper portion of the trap volume above the collection cup was gently drained by siphoning. During the siphoning, only about trap tube #7, an aliquot of the supernatant was subsampled approximately 30 cm from the top of the tube. After siphoning was complete, the upper cylinder of the trap tube was separated from the collection cup. The particle-rich water in each collection cup was pre-screened through a 500-μm-mesh sieve to remove swimmers and then mixed to disrupt large amorphous particles. The pre-screened filtrates were used for measurements of total mass flux of SPM, POC, and particulate organic nitrogen (PON) content, and HBP and EEA. All trap tubes, cartridge filters, sampling bottles, and siphon tubes were acid-cleaned before use.

### TOTAL MASS FLUX AND POC AND PON CONTENT

The pre-screened filtrate was further filtered through a pre-weighed polycarbonate membrane filter with a nominal pore size of 0.6 μm (Nuclepore, Whatman, Kent, UK) for determination of total mass flux. The filter was dried at 55°C for 48 h and weighed to within 0.1 mg. The increase in weight was defined as the total mass of SPM. The total mass flux was then estimated from the duration of trap deployment.

To determine POC and PON content, SPM in the pre-screened sample was collected onto a pre-combusted (450°C for 4 h) glass fiber filter (GF/F, Whatman). The filter was kept frozen until analysis (-30°C). The filter was fumed overnight with HCl (using the vapor method) to remove carbonates and then dried at 60°C for 24 h. POC and PON were quantified using an elemental analyzer (NA-1500, Fisons Instruments, Beverly, MA, USA).

### HETEROTROPHIC BACTERIAL PRODUCTION IN SINKING PARTICULATE MATTER

We determined HBP_SPM_ by measuring the rate of methyl-tritiated-thymidine (^3^H-TdR) incorporation into bacterial cells to measure DNA synthesis (Fuhrman and Azam, 1980). The pre-screened sample from trap tube #7 was transferred into a graduated cylinder to accurately measure the volume. ^3^H-TdR (GE Healthcare, formerly Amersham Biosciences, Buckinghamshire, UK; final concentration, 10 nmol L^−1^; specific activity, 2.70 TBq mmol^−1^) was added to 3 mL aliquots of duplicate samples and duplicate killed controls [killed by adding ice-cold TCA to a final concentration of 5% (w/v)]. The samples were incubated in the dark at the *in situ* temperatures (±2°C) of the trap deployment depths. The incubation time for determination of ^3^H-TdR incorporation rates varied between the samples both for scheduling convenience and because of the limited space in incubators. The samples were incubated for 6 h for the 50-m sample from station 44, 12 h for the 100-m sample from station 20, 17 h for the 100-m sample from station 35, and 24 h for all other samples.

Incubations were ended by adding TCA to a final concentration of 5% (w/v). After incubation, bacterial cells were collected on a 0.22-μm nitrocellulose filter (Millipore, Bedford, MA, USA). The filter was dissolved in ethyl acetate, and then mixed with liquid-scintillation cocktail (Ultima Gold). Radioactivity was determined with the liquid scintillation counter. We converted incorporation rates to HBP_SPM_ by using a conversion factor of 3.4 × 10^10^ μg-C (mol TdR)^−1^, assuming 1.7 × 10^18^ bacterial cells (mol TdR)^−1^ and a cellular carbon content of 20 fg-C cell^−1^ (Lee and Fuhrman, 1987; Simon and Azam, 1989; Simon et al., 1992).

Because we filled the trap tubes with 0.2-μm filtered seawater, we considered the measured HBP_SPM_ to be predominantly from bacteria attached to SPM, and not from either free-living bacteria or bacteria attached to suspended particles in the ambient seawater.

### EXTRACELLULAR ENZYMATIC ACTIVITIES IN SINKING PARTICULATE MATTER

To determine EEA in the SPM samples, we measured the hydrolysis rates of fluorogenic substrate analogs (Hoppe, 1983; Riemann et al., 2000). We measured not only bulk trap samples but also supernatant samples collected from the trap tubes to account for the contribution of dissolved enzymes. The EEAs in SPM were determined as the difference between bulk and supernatant activities. Eight 1.95-mL aliquots of the sieved samples, as used in the measurement of HBP_SPM_, were dispensed into disposable methacrylate cuvettes (1 cm × 1 cm path length), which were prewashed with alkaline detergent and dilute HCl. A 0.05-mL aliquot of the substrate solution was added to each duplicate aliquots to determine EEA for four classes of enzymes: LAPase activity, using L-leucine-(4-methyl-7-coumarinylamide) hydrochloride (Leu-MCA) as a substrate analog; BGase activity, using 4-methylumbelliferyl-β-D-glucoside (MUF-B-Glu) as a substrate analog; lipase activity, using 4-methylumbelliferyl oleate (MUF-O) as a substrate analog; and APase activity, using 4-methylumbelliferyl phosphate free acid (MUF-P) as a substrate analog. The fluorogenic substrate analogs were added to a final concentration of 200 μmol L^−1^.

The samples were incubated in the dark at their respective *in situ* temperatures (±2°C) for about 2 h. Concentrations of the hydrolytic products aminomethyl coumarin (AMC), liberated from Leu-MCA, and 4-methylumbelliferone (MUF), liberated from MUF-B-Glu, MUF-O, and MUF-P, were measured using a spectrofluorometer (RF-5300 PC, Shimadzu, Kyoto, Japan). Just before measurement, a 1-mL aliquot of 0.4 mol L^−1^ borate buffer solution which adjusted pH in NaOH was added to each sample to maximize fluorescent intensity; at pH 8 for AMC and pH 10 for MUF (Fukuda et al., 2000). The excitation/emission wavelengths were 380 nm/440 nm for AMC and 365 nm/448 nm for MUF. The concentrations of liberated AMC and MUF were calculated from standard curves prepared at each station. All substrate analogs and standards were purchased from Sigma-Aldrich (St Louis, MO, USA).

Although the durations of the incubations in this study were relatively short (approximately 2 h), we did not verify the linearity of the assay. Substrate depletion was never apparent, because the fraction of added substrate that was hydrolyzed never exceeded 0.6%. Thus, the change in substrate concentration and saturation with time during incubation was considered negligible. Note that the use of dissolved substrates to track EEAs on SPM might yield underestimates, as with HBP_SPM_ measurements.

### STATISTICAL ANALYSIS

We performed Pearson product-moment correlation analysis between several parameters measured in the sediment trap samples and in the seawater samples. We tested for normal distributions and constant variances with the linear regression models. We also carried out Spearman’s rank-order correlation, a non-parametric analysis that does not require a normal distribution or homoscedasticity of the variables. For statistical analysis, we used the program Sigmastat, included in the Sigmaplot 11 software package (Systat Software, Chicago, IL, USA). We used a significance level of *P* < 0.05.

### REAGENTS

All reagents used in these and other analyses were of analytical grade unless otherwise specified.

## RESULTS

### HYDROGRAPHIC CONDITIONS AND NUTRIENT AND Chl *a* DISTRIBUTIONS

**FIGURE 2:**
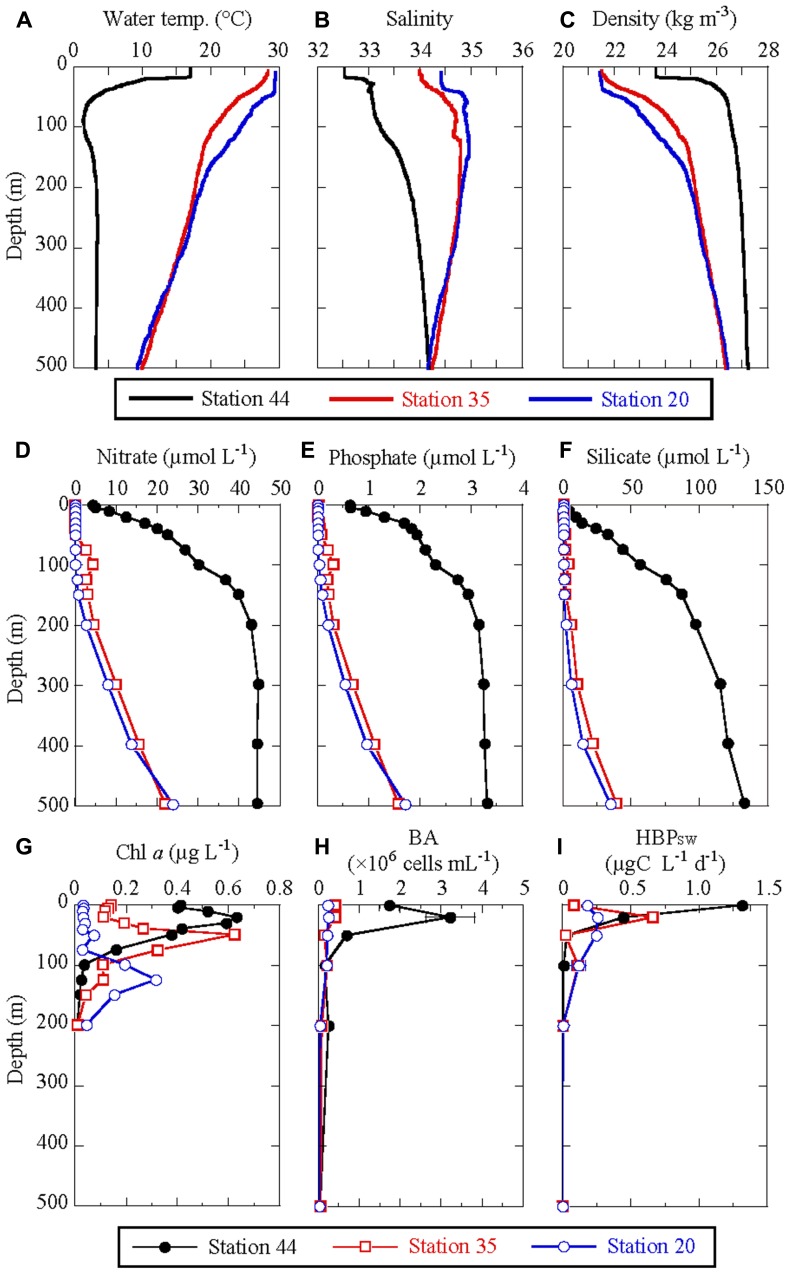
**Depth profiles of physical and chemical parameters in the water column at three stations in the western North Pacific Ocean. (A)** water temperature, **(B)** salinity, **(C)** density (sigma-t), **(D)** nitrate concentration, **(E)** phosphate concentration, **(F)** silicate concentration, **(G)** Chl *a* concentration, **(H)** BA, and **(I)** HBP_SW_. Error bars in **(H)** indicate the standard deviation of triplicate measurements. Error bars in **(I)** indicate half the difference between two replicates. BA, bacterial abundance; HBP_SW_, heterotrophic bacterial production in seawater.

Station 44, located in the subarctic region, was characterized by low water temperature and salinity, and a strong thermocline (**Figures 2A–C**). The mixed layer depth was 18 m, where the water temperature ranged between 15 and 17°C. Temperature decreased sharply to a minimum of 1.4°C at 90 m, and then remained constant at around 3.3°C from 200 to 500 m. Station 35, located in the Kuroshio Extension area, and station 20, located in the NPSG, exhibited similar water-column structures. Surface water temperature was around 29°C and decreased gradually to about 10°C at 500 m. A closer look shows a mixed layer in the upper 50 m at station 20, whereas the water column was stratified throughout the upper 500 m at station 35. The hydrographic profiles were extremely similar to 165°E transects of hydrographic atlas of the World Ocean Circulation Experiment (WOCE; Telley, 2007).

There were considerable differences in depth profiles of dissolved nutrients between the nutrient-replete subarctic region and the oligotrophic Kuroshio Extension region and the NPSG **Figures 2D–F**). Both N and P were replete at station 44, even at the surface, where concentrations of nitrate and phosphate were 4.4 and 0.62 μmol L^−1^, respectively, increasing rapidly with depth to 40 and 3 μmol L^−1^, respectively, at 200 m. The depth profile of silicate at station 44 shows slightly lower concentrations (≤10 μmol L^−1^) in the surface productive layer (0–30 m), with a silicate:nitrate ratio less than one. Like nitrate and phosphate, silicate concentrations increased with depth and reached 130 μmol L^−1^ at 500 m. At stations 35 and 20, dissolved nutrients were depleted in the upper water column. Concentrations of nitrate, phosphate, and silicate above the Chl *a* maximum layer (described below) were at or below the detection limits. The concentrations then increased gradually with depth and reached 22–24 μmol L^−1^ for nitrate, 1.6–1.7 μmol L^−1^ for phosphate, and 35–40 μmol L^−1^ for silicate at 500 m. The depth profiles of dissolved nutrients were also extremely similar to 165°E transects of hydrographic atlas of WOCE (Telley, 2007).

The maximum concentrations of Chl *a* were comparable between stations 44 and 35 (0.62 and 0.63 μg L^−1^, respectively; **Figure 2G**). However, the depth of the Chl *a* maximum layer at station 44 (20 m) was shallower than at station 35 (50 m). Station 20 exhibited lower Chl *a* concentrations, with the maximum of 0.32 μg L^−1^ observed in the deep layer at 125 m. Depth-integrated Chl *a* biomass in the euphotic zone was highest at station 44 (26.3 mg m^−2^), followed by stations 35 (14.7 mg m^−2^) and 20 (10.3 mg m^−2^; **Table 2**). Imai et al. (2002) reported the depth-integrated Chl *a* biomass at station 44 was 17–64 mg m^−2^. Suzuki et al. (1997) reported it was 35, 9, 7, and 6 mg m^−2^ at 44°N, 33°N, 24°N, and 16°N along the 175°E transect, respectively. Our data was within the range or similar to previous reports.

**Table 2:** Depth-integrated production and flux estimates from sediment trap deployments at three stations in the North Pacific Ocean.

Station	IPP^a^ (mg C m^−2^ day^−**1**^)	HBP_SW_^b^ (mg C m^−2^ day^−1^)	HBP_SPM_^c^ (mg C m^−2^ day^−1^)	POC flux at shallow layer^d^ (mg C m^−2^ day^−1^)	POC flux at deep layer^e^ (mg C m^−2^ day^−1^)
44	373	27.1 (0.07)	0.03 (0.0001)	116.9 (0.31)	67.1 (0.18)
35	195	28.0 (0.14)	0.92 (0.005)	31.9 (0.16)	18.5 (0.09)
20	186	28.7 (0.15)	0.57 (0.003)	25.9 (0.14)	6.68 (0.04)

*Values in parentheses are the proportion of IPP.*

a *Integrated primary production from the surface to the euphotic depth*.

b *Heterotrophic bacterial production in seawater integrated from the surface to 500 m*.

c *Heterotrophic bacterial production in suspended particulate matter integrated from 50 to 500 m at station 44, and from 100 to 500 m at stations 35 and 20*.

d *50 m at station 44 and 100 m at stations 35 and 20*.

e *500 m at all stations*.

Light–depth profiles and PP values at the study sites were provided courtesy of Dr K. Suzuki of Hokkaido University, Japan (personal communication). PP was measured using *in situ* bottle incubations based on ^13^C-bicarbonate incorporation. The highest rate of PP was 15.5 mg C m^−3^ day^−1^ at 5-m depth at station 44 (data not shown). The depth-integrated PP in the euphotic zone (IPP) at station 44 (373 mg C m^−2^ day^−1^) was nearly double that at stations 35 and 20 (195 and 186 mg C m^−2^ day^−1^, respectively; **Table 2**). Hama (1997) reported IPP is 350–410, 220–600, and 180–220 mg C m^−2^ day^−1^ at subarctic Pacific, Kuroshio region, and subtropical Pacific, respectively. IPP of this study were within the range of previous reports.

### HETEROTROPHIC BACTERIAL CELL ABUNDANCE AND PRODUCTION IN SEAWATER

At all stations, the maximum BA was at 20 m (**Figure 2H**). At station 44 (20 m) BA was 3.22 × 10^6^ cells mL^−1^, which is particularly high relative to BA at stations 35 and 20 (0.42 × 10^6^ and 0.25 × 10^6^ cells mL^−1^, respectively). BA decreased rapidly with depth, with comparable values for all three stations at 100 m of around 0.2 × 10^6^ cells mL^−1^.

**Figure 2I** shows depth profiles of HBP_SW_. The highest rate, 1.31 μg-C L^−1^ day^−1^, was found at the surface at station 44 and decreased rapidly with depth. Maximum rates at stations 35 and 20 were 0.66 and 0.26 μg-C L^−1^ day^−1^, respectively, observed in the subsurface layer (20 m). We calculated the bacterial-cell-specific rate of HBP_SW_ using BA and the bulk-community rate data. The distribution of the cell-specific rate (data not shown) was distinguishable from that of the bulk community rate. The cell-specific HBP_SW_ was highest at 1.59 fg-C cell^−1^ day^−1^ at station 35 (20 m). The maximum cell-specific rates at stations 44 and 20 were 0.76 fg-C cell^−1^ day^−1^ at 0 m and 1.11 fg-C cell^−1^ day^−1^ at 50 m, respectively.

### TOTAL MASS FLUX AND POC FLUX

**FIGURE 3:**
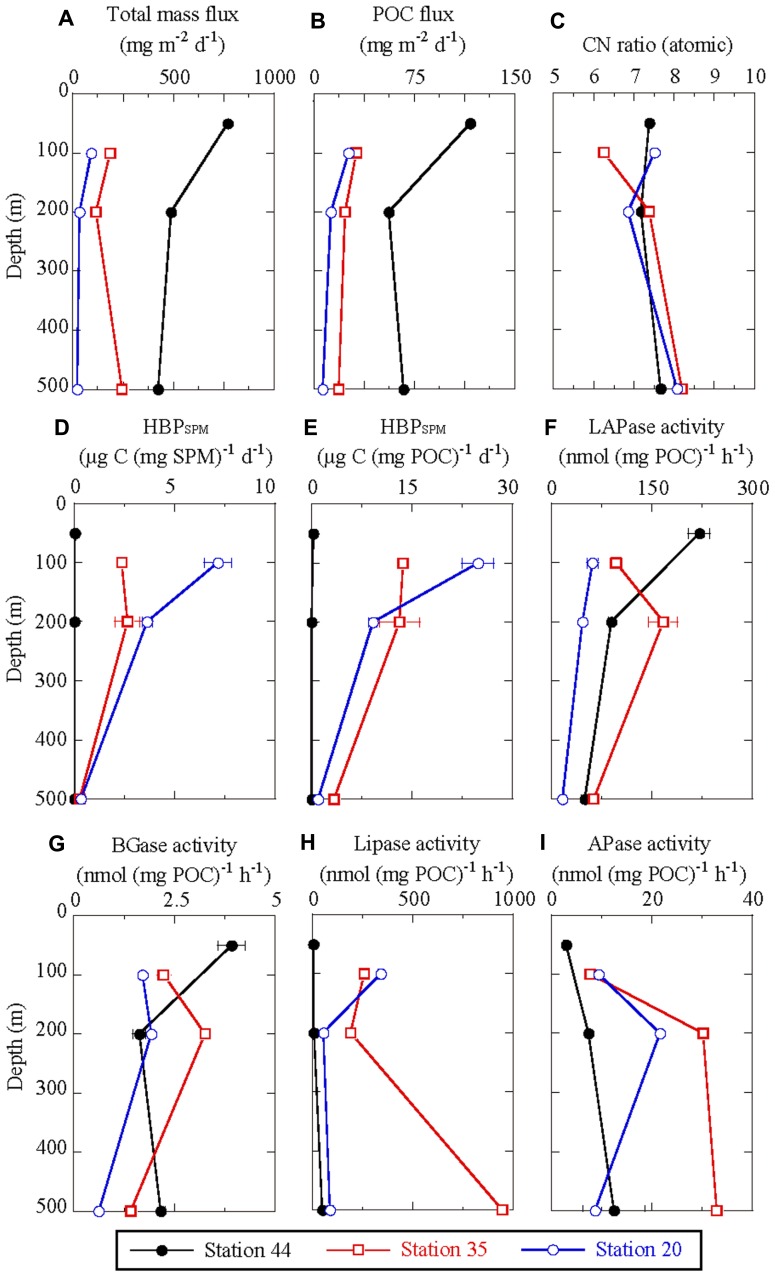
**Vertical changes in (A)** total mass flux of suspended particulate matter (SPM), **(B)** POC flux, and **(C)** C:N ratio, as determined from sediment trap experiments. **(D,E)** Depth profiles of HBP_SPM_ determined using ^3^H-TdR incorporation, normalized to SPM dry-weight **(D)** and POC **(E)**. **(F–I)** Activity depth profiles of **(F)** LAPase, **(G)** BGase, **(H)** lipase, and **(I)** APase in SPM, normalized to POC. Error bars in **(D–I)** indicate the range of duplicate analyses. Symbols and abbreviations are the same as in **Figure 2**.

We were able to collect 0.3–6 mg dry weight SPM from the sediment trap tubes at each sampling depth (**Table 1**). The total mass flux calculated from the SPM weight was considerably higher at station 44 than at stations 35 and 20 (**Figure 3A**). The total mass flux was between 424 and 771 mg m^−2^ day^−1^ at station 44, 116–224 mg m^−2^ day^−1^ at station 35, and 19–90 mg m^−2^ day^−1^ at station 20.

Depth profiles of POC flux were similar to those of total mass flux (**Figure 3B**). POC flux at 500 m was 67.1, 18.5, and 6.68 mg C m^2^ day^−1^ at stations 44, 35, and 20, respectively. Depth profiles of PON flux were similar to those of POC flux (data not shown). Although the single measurement of POC and PON content at each depth precluded statistical analysis, the C:N ratio at station 44 seemed to be constant throughout all depths, whereas at stations 35 and 20 the ratio seemed to increase with depth (**Figure 3C**). The increase in the C:N ratio between 200 and 500 m was 0.49, 0.81, and 1.22 at stations 44, 35, and 20, respectively.

### HETEROTROPHIC BACTERIAL PRODUCTION IN SINKING PARTICULATE MATTER

There was a wide range in the incubation times for HBP_SPM_ measurements in this study (6–24 h). The fraction of added ^3^H-TdR incorporated into bacterial cells never exceeded 3%. This suggests that prolonged incubation would cause only small changes in the degree of substrate saturation. The dependence of ^3^H-TdR incorporation on ^3^H-TdR concentration is reportedly relatively small (Bjørnsen and Kuparinen, 1991). However, we did not verify the linearity of the HBP_SPM_. There is a potential fourfold error in HBP_SPM_ estimates if the linearity between incubation time and ^3^H-TdR incorporation lasts for only 6 h.

We assumed that the contents of trap tubes at each sample depth were homogeneous across replicate tubes, and estimated HBP_SPM_ normalized to the SPM dry weights determined as described above (**Figure 3D**). Because the total amount of SPM collected at each depth was very small (0.3–6 mg) with a 0.1-mg resolution, weight-specific rates could involve large errors, especially in the samples with the smallest amounts of SPM. Therefore, we also calculated POC-normalized rates of HBP_SPM_, because of the higher precision and sensitivity of POC analysis when the measured POC is between 100 and 450 μg C and the detection limit is 2 μg C (Nieuwenhuize et al., 1994). Depth profiles of weight-specific and POC-specific HBP_SPM_ resemble each other (**Figures 3D,E**); thus the two calculations yield internally consistent results. Henceforth all HBP_SPM_ values are reported as POC-specific values unless otherwise stated.

Although HBP_SW_ was relatively comparable among the stations (**Figure 2I**), we found exceptionally low HBP_SPM_ at subarctic station 44 relative to those at stations 35 and 20. At station 44, HBP_SPM_ was 0.31 μg-C (mg POC)^−1^ day^−1^ at 50 m, decreasing with depth to 0.01 μg-C (mg POC)^−1^ day^−1^ at 500 m. At stations 35 and 20, HBP_SPM_ was 13.7 and 24.9 μg-C (mg POC)^−1^ day^−1^ at 100 m, respectively, two orders of magnitude higher than at station 44. The HBP_SPM_ decreased with depth to 3.4 and 0.9 μg C (mg POC)^−1^ day^−1^ at 500 m at stations 35 and 20, respectively; these values are still higher than the surface maximum value at station 44.

### EXTRACELLULAR ENZYME ACTIVITY IN SINKING PARTICULATE MATTER

In this study, we minimized enzyme activities derived from bacterial cells or suspended particles in ambient seawater by filling the trap tubes with pre-filtered (0.2 μm) seawater before deployment. The percentages of EEAs in the dissolved fraction (<0.2 μm) were 0–16.1% for LAPase, 0–17.8% for BGase, 0–10.8% for lipase, and 0–37.1% for APase. These estimates of dissolved EEA in the seawater used for filling the tubes are probably maximum estimates because we assayed the supernatant from the trap tubes, but not the activity in seawater used for filling before trap deployment.

**FIGURE 4:**
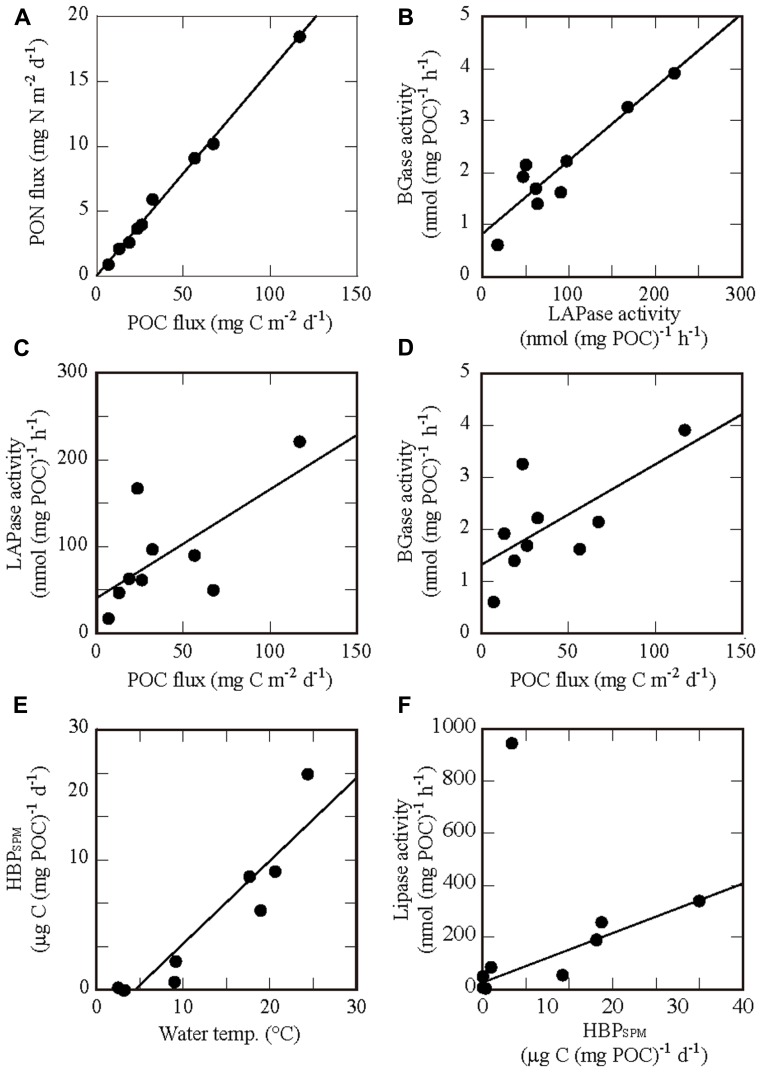
**Relationships between physical, chemical and biological variables in seawater and SPM for which significant correlations were found (Pearson product-moment correlation, *P* < 0.05). (A)** PON flux vs POC flux, **(B)** BGase activity vs LAPase activity, **(C)** LAPase activity vs POC flux, **(D)** BGase activity vs POC flux, **(E)** HBP_SPM_ vs water temperature, and **(F)** Lipase activity vs HBP_SPM_. The regression line in **(F)** was determined after excluding one outlying data point.

Depth profiles of EEA in SPM normalized to POC are shown in **Figures 3F–I**. As with HBP_SPM_, profiles of EEAs normalized to POC are similar to those normalized to SPM dry-weight (not shown). Although LAPase activities were two orders of magnitude higher than BGase activities, the profiles of these two enzyme activities were very similar. As described in Section “Relationships Between Measurements in Sinking Particulate Matter and Seawater” and shown in **Figure 4**, there was a significant linear relationship between the activities of LAPase and BGase, with a slope (BGase activity/LAPase activity) of 0.027 ± 0.001 (*n* = 9). Depth profiles and geographic variations of lipase and APase activities were distinctive, and different from those of LAPase and BGase activities (**Figures 3H,I**). Lipase activity at station 44 was low at all depths and exceptionally high at 500 m at station 35. Depth profiles of APase activity at stations 35 and 20 were similar to those of BGase activity, but distinctly different at station 44, where APase activity increased with depth.

### RELATIONSHIPS BETWEEN MEASUREMENTS IN SINKING PARTICULATE MATTER AND SEAWATER

We performed Pearson product-moment correlation analysis between several variables measured in the sediment trap samples (HBP_SPM_, EEA, POC flux, PON flux, and C:N ratio) and in the seawater samples [water temperature and HBP_SW_ at trap depths at the three stations (*n* = 9)]. POC-normalized HBP_SPM_ and EEA in SPM were used in the analyses. The results of regression analysis are shown in **Table 3**, and *x*–*y* plots for some of the relationships with significant correlations are shown in **Figure 4**, along with regression lines.

**Table 3:** Pearson product-moment correlation matrix comparing variables measured in SPM (HBP_SPM_, EEA, POC and PON fluxes, and C:N ratio) and in seawater (water temperature and HBP_SW_).

	LAPase	BGase	Lipase	APase	POC flux	PON flux	C:N ratio	Water temp.	HBP_SW_
HBPSPM	–0.032	0.040	0.201	0.113	–0.415	–0.397	–0.399	0.931	0.764
	0.935	0.919	0.603	0.773	0.266	0.290	0.372	<0.001	0.017*
LAPase		0.926	–0.190	–0.089	0.726	0.736	–0.200	–0.171	0.052
		<0.001	0.624	0.820	0.027	0.024	0.605	0.659	0.895
BGase			–0.280	–0.044	0.687	0.695	–0.318	–0.085	0.069
			0.466	0.910	0.041	0.038	0.404	0.829	0.861
Lipase				0.623	–0.401	–0.416	0.394	0.211	0.097
				0.073	0.285	0.265	0.294	0.586	0.861
APase					–0.513	–0.540	0.336	0.236	–0.407
					0.158	0.133	0.376	0.540	0.277
POC flux						0.998	–0.087	–0.626	–0.028
						<0.001	0.825	0.071	0.944
PON flux							–0.147	–0.600	0.010
							0.706	0.088	0.980
C:N ratio								–0.419	–0.501
								0.262	0.170
Water temp.									0.651
									0.058

*HBP_SPM_ and EEAs in SPM were normalized to POC. Upper and lower numbers indicate the correlation coefficient (r) and the P value, respectively (n = 9). Significant correlations (P < 0.05) are underlined.*

* Failed constant variance (homoscedasticity) test. SPM, sinking particulate matter; HBP_SPM_, heterotrophic bacterial production on SPM; EEA, extracellular enzyme activity; POC, particulate organic carbon; PON, particulate organic nitrogen; HBP_SW_, heterotrophic bacterial production in seawater.

We found significant correlations (*P* < 0.001) between POC flux and PON flux. This is consistent with the result that C:N ratios in SPM were relatively constant among sampling stations and depths (**Figure 3C**). Among the four types of extracellular enzymes, we found a significant correlation only between LAPase activity and BGase activity. There were also significant correlations between the activities of these two enzymes and both POC flux and PON flux. HBP_SPM_ was correlated significantly with water temperature, suggesting temperature dependency of heterotrophic bacterial activity in SPM. Conversely, no significant correlation was found between HBP_SW_ and water temperature. The regression analysis between HBP_SPM_ and HBP_SW_ returned a low *P* value (0.017), suggesting a significant correlation. However, the data for these rates did not meet the common assumption of constant variance (homoscedasticity) for standard parametric statistical tests.

There was no significant correlation between HBP_SPM_ and the activities of any of the enzymes tested (**Table 3**). However, non-parametric Spearman’s rank-order correlation analysis revealed a significant correlation between HBP_SPM_ and lipase activity (ρ = 0.733, *P* = 0.020, *n* = 9). Furthermore, after excluding the data from 500 m at station 35, where an exceptionally high lipase activity was observed (**Figure 3H**), there was a significant linear correlation between these two variables (*r* = 0.926, *P* < 0.001, *n* = 8; **Figure 4F**). Lipase activity, excluding the outlier, also showed a good correlation with water temperature (ρ = 0.905, *P* < 0.001, *n* = 8). It is uncertain whether lipase activity was regulated by water temperature directly, by heterotrophic bacterial activity, or by both.

## DISCUSSION

### PRIMARY AND HETEROTROPHIC PRODUCTION

In this study, we investigated a range of chemical and biological variables related to transport and biological utilization of organic matter in upper ocean environments in the western North Pacific. By coupling the IPP data with our estimates of SPM flux and heterotrophic bacterial activity, we were able to examine the fate and budget of organic matter in the upper ocean at a range of geographic sites in the North Pacific Ocean. We also obtained other parameters related to the production and flux of organic carbon in the systems studied, including depth-integrated HBP_SW_ from the surface to 500 m, depth-integrated HBP_SPM_, and the export flux of POC at two depth intervals (**Table 2**).

Although water-column BA was highly variable between the stations (**Figure 2H**), we obtained comparable values for depth-integrated HBP_SW_, ranging from 27.1 to 28.7 mg C m^−2^ day^−1^ based on the ^3^H-Leu incorporation rate (**Table 2**). This similarity probably results from the combined effects of BA, thickness of the high productivity layer, and cell-specific production rates. Station 44 exhibited higher biomass but a shallow productive layer depth and lower cell-specific production. In contrast, station 20 had lower BA but a relatively thick layer of production and considerably higher cell-specific productivity. Station 35 exhibited characteristics between those of the other two stations. Van Wambeke et al. (2008) determined depth-integrated HBP_SW_ in the eastern South Pacific Ocean and in the upwelling region off the coast of Chile. The integrated HBP_SW_ values that we observed in the northwest Pacific Ocean in this study were relatively low compared to those reported in the oligotrophic South Pacific gyre (43 mg C m^−2^ day^−1^), and substantially lower than those found in the upwelling region off Chile (392 mg C m^−2^ day^−1^).

Steinberg et al. (2008) calculated depth-integrated BCD in seawater and the ratio of these BCD to loss of POC flux in the subarctic northwest Pacific Ocean and NPSG near Hawaii. We performed rough calculation of depth-integrated BCD with bacterial growth efficiency of 0.15 (Steinberg et al., 2008). The depth-integrated BCD and the ratio of BCD to loss of POC flux at station 44 (50–500 m) was 15.6 mg m^−2^ day^−1^ and 318.7%. This value was lower than reported value in the subarctic northwest Pacific Ocean (54.3–163.0 mg m^−2^ day^−1^ and 524–1573%, 150–500 m) of Steinberg et al. (2008). It was suggested that the difference was caused by wide range seasonal change of POC flux at the subarctic site (Steinberg et al., 2008). The depth-integrated BCD and the ratio of BCD to loss of POC flux at stations 35 and 20 (100–500 m) was 13.4 and 19.2 mg m^−2^ day^−1^ and 339.1 and 260.6%, respectively. These values were similar to reported value in NPSG near Hawaii (18.7–61.3 mg m^−2^ day^−1^ and 138–435%, 150–500 m; Steinberg et al., 2008).

To determine the ratio of carbon incorporated by heterotrophic bacteria in seawater to that produced by phytoplankton in the euphotic zone, we calculated the ratio of integrated-HBP_SW_ to IPP (**Table 2**). Our ratios (0.07–0.15) are comparable to the data summarized by Ducklow (2000). The ratio from station 44 in the subarctic gyre was similar to those from the subarctic (0.09 in the North Pacific; Kirchman et al., 1993) and Antarctic (0.04 in Ross Sea; Carlson et al., 1998), and the ratios at stations 35 (Kuroshio Extension area) and 20 (subtropical gyre) were similar to other data from equatorial and subtropical oceans.

In addition to estimates of HBP_SW_, we were able to approximate the contribution of HBP_SPM_ to the carbon budget in the upper water column. We estimated HBP_SPM_ from rates of ^3^H-TdR incorporation in SPM samples by using a conversion factor for carbon bacterial production (see Heterotrophic Bacterial Production in Sinking Particulate Matter). We calculated depth-integrated values from HBP_SPM_ normalized to POC [μg-C (mg POC)^−1^ day^−1^] and fluxes of POC (mg-POC day^−1^), with an assumed particle sinking rate of 100 m day^−1^ (e.g., McDonnell and Buesseler, 2010). In contrast to the depth-integrated HBP_SW_, which was almost identical among the stations, the depth-integrated HBP_SPM_ exhibited considerable variation (**Table 2**). We found a particularly low value of 0.03 mg C m^−2^ day^−1^ at station 44, which corresponded to only 0.01% of IPP. The values at stations 35 and 20 were higher at 0.92 and 0.57 mg C m^−2^ day^−1^, respectively. The ratio of HBP_SPM_ to IPP was very low, <0.5%, compared to the same ratio for HBP_SW_. However, a direct comparison of depth-integrated HBP_SPM_ and depth-integrated HBP_SW_ might be unreasonable, because the former did not include HBP above the depth of the uppermost trap deployment, which is almost equivalent to the euphotic zone and potentially the most productive as indicated by the vertical profiles of HBP_SW_ (**Figure 2I**). Therefore, it might be more reasonable to compare depth-integrated HBP_SPM_ with the POC flux at the depth of shallowest trap deployment (**Table 2**), which would correspond to the POC input to the water column where depth-integrated HBP_SPM_ was estimated. At stations 35 and 20, depth-integrated HBP_SPM_ was 2.9 and 2.2% of the POC input, respectively, whereas it was only 0.03% at station 44. In contrast, depth-integrated HBP_SW_ was 23, 88, and 111% of the POC input to the water column at stations 44, 35, and 20, respectively. This suggests a high contribution of bacterial incorporation to the dissolution of POC at stations 35 and 20. At station 44, the contribution from heterotrophic bacteria was relatively low. However, LAPase and BGase activity in SPM was relatively high at station 44 (**Figures 3F,G**). This suggests that the dissolution process, converting POC to DOC, was more active than the incorporation process.

The considerable difference between HBP_SPM_ at station 44 and at other stations is explainable by temperature regulation of bacterial activity. Water temperature has been shown to regulate heterotrophic bacterial abundance, production, and growth rate in marine environments (Shiah and Ducklow, 1994a,b; Pomeroy and Wiebe, 2001).

### CARBON EXPORT FLUX

The POC flux was highest by a substantial amount at station 44, and higher at station 35 than at station 20 (**Table 2**); the same order as for depth-integrated Chl *a* biomass and IPP. This suggests that phytoplankton-derived materials were the major source of SPM collected by sediment traps. The export ratios (*e*-ratio = POC flux/IPP) were estimated at 0.18, 0.09, and 0.04 at stations 44, 35, and 20, respectively. A relatively large amount of organic matter produced in the surface euphotic zone was efficiently exported to the mesopelagic zone at the subarctic station compared to the station in the subtropical gyre. Sinking particles are known to lose nitrogen faster than carbon because of preferential degradation of organic nitrogen, thus increasing their C:N ratio with depth (Suess, 1980; Martin et al., 1987). The observed constancy of C:N ratios at station 44, in contrast to those at stations 35 and 20, which increased with depth, indicates that the extent and rate of organic matter degradation in SPM was relatively low at station 44.

A previous study found diatoms to be the dominant phytoplankton species at station 44 (Suzuki et al., 2002). The biogenic silica (BSi) in SPM collected from the same sediment traps as used in our study (data not shown; Dr H. Saito, personal communication) showed that the flux of BSi was highest at station 44 (14 times that at station 35 and 66 times that at station 20, at 500 m). These data indicate that the source of SPM at station 44 was predominantly diatoms; the relatively low silicate:nitrate ratio in the upper layers at the subarctic station suggests a higher abundance of diatoms in the phytoplankton community.

### ROLE OF EXTRACELLULAR ENZYMES IN CARBON FLUX

Previous studies have used two approaches to evaluate EEA in SPM. Lower concentrations of substrate analogs, from 0.02 to 10 μmol L^−1^, were used to determine hydrolysis rates (*v*) around the presumed *in situ* level of naturally occurring substrates (Karner and Herndl, 1992; Smith et al., 1992; Hoppe et al., 1993; Vetter and Deming, 1994; Taylor et al., 2009). In contrast, much higher concentrations in excess of substrate saturation (approximately 200 μmol L^−1^) were added to samples used for estimating the maximum potential hydrolysis rates (*V*_max_; Hoppe et al., 1993; Huston and Deming, 2002; this study). Because of the number and volume of samples required to maintain *in situ* conditions, and the limitations of time and space, we were unable to conduct a kinetic assay of enzyme reactions using multiple concentrations of substrate analogs, although this approach has been proposed (Hoppe, 1993; Sebastián and Niell, 2004; Suzumura et al., 2012) and could provide the kinetic parameters of enzymatic reactions, including the half-saturation constant (*K*_m_). Given that the substrate saturation experiments of Huston and Deming (2002) determined that *K*_m_ was 100 μmol L^−1^ for LAPase and 30 μmol L^−1^ for BGase, the concentrations of fluorescent substrates used in this study (200 μmol L^−1^) are probably at or above saturation, and hence the hydrolysis rates determined can be regarded as *V*_max_. For APase activities, Suzumura et al. (2012) reported that high concentrations of MUF-P (approximately 1000 nmol L^−1^) did not inhibit APase activities in open-ocean samples, although Steen and Ziervogel (2012) point out that high levels of substrate can inhibit APase activity. Note also that the use of dissolved substrates to track EEAs on SPM could yield underestimates.

We obtained a significant correlation between the activities of LAPase and BGase, with a BGase:LAPase activity ratio (slope) from linear regression analysis of 0.027. Taylor et al. (2009) also found a significant correlation between LAPase and BGase activities in SPM from the Cariaco Basin, Venezuela (Pearson product-moment correlation, *P* < 0.0001). Regardless of a significant correlation, BGase activity has been reported lower by a factor of 10–1000 than that of LAPase in SPM and marine snow in a range of ocean environments (Smith et al., 1992; Huston and Deming, 2002; Taylor et al., 2009; this study). The notably low BGase:LAPase activity ratio suggests that proteins (nitrogen-rich compounds) are solubilized faster than polysaccharides (carbon-rich compounds) through enzymatic hydrolysis, resulting in the observed increase in C:N ratio of SPM with depth (**Figure 3C**).

Studies on the activity of marine lipases are rare (Martinez et al., 1994; Vetter and Deming, 1994), in comparison with those of LAPase, BGase and APase. Among the EEAs tested in this study, only lipase exhibited a significant correlation with HBP, if one outlier was excluded; thus the temperature dependency of lipase activity is consistent with that of HBP.

The temperature dependence of hydrolytic enzymes in marine environments is not straightforward. Vetter and Deming (1994) found that the central tendency of Q_10_-values, that is, the slope of an Arrhenius plot derived from EEA and temperature, varied greatly between different enzymes and different samples of surface sediments and SPM from the Northeast Water Polynya near Greenland. Christian and Karl (1995) found that relative activities of LAPase and BGase in seawater varied widely among three oceanic regions: the subtropical North Pacific, the equatorial Pacific, and the Southern Ocean, with LAPase:BGase (*V*_max_/*V*_max_) ratios between 0.13 and 1052. It is possible that the temperature responses of enzymes reflect important differences between dominant bacterial communities.

Because previous studies observed increasing C:P and N:P ratios in SPM as a function of depth (Martin et al., 1987; Christian et al., 1997), a rapid loss of phosphorus through selective remineralization is considered an important process in the phosphorus biogeochemical cycle (Karl et al., 2012). Enzymatic hydrolysis by APase is arguably the most important remineralization pathway for phosphorus, one of the nutrients essential for marine productivity, because APase is found in a wide variety of eukaryotic phytoplankton and in both autotrophic and heterotrophic prokaryotes (Cembella et al., 1982; Hoppe, 2003). APase activity is usually regulated by phosphate supply, as the activity increases with decreasing phosphate concentration (Cembella et al., 1982). Therefore, APase activity is considered a useful indicator of phosphate deficiency in seawater (Van Wambeke et al., 2002; Dyhrman and Ruttenberg, 2006; Lomas et al., 2010; Suzumura et al., 2012). In the present study, we found no significant correlation between APase activity in SPM and phosphate concentration in seawater by Pearson product-moment correlation or Spearman rank-order correlation (*P* > 0.05). Phosphate concentrations at the depth of the shallowest trap deployment varied substantially, between the detection limit at station 20 and 1.9 μmol L^−1^ at station 44 (**Figure 2E**). However, there was no substantial difference in APase activity between these stations (**Figure 3I**). For microbes thriving in SPM, APase activity might not be directly affected by the phosphate-replete or depleted conditions in the surrounding waters.

## CONCLUSION

This study demonstrates the relative difference in microbial activities on SPM at a range of geographic sites. Despite considerable differences in primary productivity, HBP_SW_ was relatively consistent among the stations, whereas HBP_SPM_ was substantially lower at the subarctic station. In the oligotrophic subtropical and Kuroshio Extension regions, total HBP, that is, the sum of HBP_SW_ and HBP_SPM_, was comparable to the POC export flux. In contrast, the POC export flux greatly exceeded HBP at the subarctic station. This is most likely due to a temperature dependency of heterotrophic bacterial activity. Differences in source materials, such as diatom-dominated particles, have been proposed as an important factor controlling the export efficiency of SPM. Furthermore, a kinetic approach would provide more detailed information on *in situ* EEAs and their contribution to organic matter degradation in SPM. More research into SPM biogeochemistry is necessary to identify the role of SPM in oceanic organic carbon cycling.

## Conflict of Interest Statement

The authors declare that the research was conducted in the absence of any commercial or financial relationships that could be construed as a potential conflict of interest.
